# Analysis of Prognostic Factors Affecting Quality of Life After Ischemic Stroke

**DOI:** 10.3390/jcm15093471

**Published:** 2026-05-01

**Authors:** Edyta Laska, Elżbieta Musz, Marcin Skrok

**Affiliations:** 1 Faculty of Health Sciences, Collegium Medicum, Andrzej Frycz Modrzewski Krakow University, 1 Gustaw Herling-Grudziński Street, 30-705 Krakow, Poland; 2 University Hospital in Krakow, 11 Marii Orwid Street, 30-688 Krakow, Poland; emusz@su.krakow.pl; 3 Nowy Szpital w Świeciu, 126 Wojska Polskiego Street, 86-100 Świecie, Poland; statystyka@statystyka.org.pl

**Keywords:** ischemic stroke, prognostic factors, quality of life, functional independence, social support, disease acceptance

## Abstract

**Background:** Ischemic stroke remains a major cause of disability and reduced quality of life (QoL). This study aimed to identify factors associated with QoL after ischemic stroke, with particular emphasis on independence, illness acceptance, social support, comorbidity status, and the timeliness of diagnosis and treatment. **Methods:** This single-center cross-sectional study included 100 consecutively recruited patients after ischemic stroke hospitalized in the Department of Neurology with the Stroke Unit at the S. Żeromski Specialist Hospital in Krakow. Data were collected using an author-designed questionnaire and standardized instruments: the World Health Organization Quality of Life-BREF (WHOQOL-BREF), the Multidimensional Scale of Perceived Social Support (MSPSS), the Lawton Instrumental Activities of Daily Living Scale (IADL), and the Acceptance of Illness Scale (AIS). Statistical analysis included Spearman’s rank correlation coefficient and the Mann–Whitney U, Friedman, and Kolmogorov–Smirnov tests. **Results:** Significant positive correlations were found between all WHOQOL-BREF domains and IADL, AIS, and MSPSS scores. The strongest correlations were observed between IADL and the physical and psychological QoL domains. A strong positive correlation was also found between IADL and AIS (rho = 0.88; *p* < 0.001). Better QoL and greater independence were observed in patients with fewer comorbidities. Patients who received timely diagnosis and treatment achieved better outcomes in terms of QoL, IADL, and AIS. Perceived social support was comparable across MSPSS subscales (*p* = 0.56) but positively correlated with all QoL domains (rho = 0.55–0.64; *p* < 0.001). **Conclusions:** Better QoL after ischemic stroke was associated with greater independence, higher illness acceptance, stronger perceived social support, and timely diagnosis and treatment, suggesting that post-stroke QoL is related to both functional and psychosocial factors.

## 1. Introduction

Stroke remains a major cause of death and disability worldwide and continues to represent a substantial public health burden [1]. According to the Global Burden of Disease (GBD) study, it ranks as the second leading cause of death and the third leading contributor to death and disability combined globally [1,2]. From a clinical perspective, stroke is an acute cerebrovascular disorder caused either by rupture of a cerebral blood vessel or by interruption of cerebral blood flow, both of which result in brain injury and neurological dysfunction [3]. Ischemic stroke, which accounts for approximately 80–85% of all stroke cases, is the most common type of stroke and is frequently associated with long-term neurological deficits that impair everyday functioning and reduce quality of life (QoL) [3,4,5].

QoL, as defined by the World Health Organization (WHO), refers to an individual’s perception of their position in life within the context of the culture and value systems in which they live, and in relation to their goals, expectations, standards, and concerns [6]. In stroke survivors, QoL encompasses physical functioning, psychological well-being, social participation, autonomy, and perceived availability of support. Accordingly, increasing attention has been directed toward patient-reported outcomes, particularly the perceived impact of symptoms, disability, and treatment on everyday life and well-being [7,8]. For this reason, post-stroke outcomes should be considered not only in relation to neurological deficits but also in relation to functional, psychological, and social factors.

Recovery after stroke is typically prolonged and multidimensional, requiring sustained rehabilitation and coordinated follow-up care. Population ageing and improved survival after stroke have contributed to a growing number of individuals living with post-stroke sequelae who require specialized care, rehabilitation, and long-term support. Despite substantial progress in acute stroke treatment, many patients continue to experience persistent cognitive, sensory, motor, and emotional impairments after hospital discharge. Stroke may result in cognitive, sensory, motor, and emotional disturbances that limit patients’ ability to perform daily activities and restrict their participation in social and professional life [4,9]. Motor and balance disorders, including gait impairment and increased risk of falls, may further reduce independence, whereas chronic post-stroke consequences may contribute to anxiety, depression, and other mental health problems [10]. The severity and pattern of motor, sensory, and cognitive impairment after stroke vary considerably between patients, contributing to heterogeneity in clinical presentation and QoL outcomes [9].

Health professionals play an important role in improving stroke awareness, promoting secondary prevention, and strengthening adherence to therapeutic recommendations after hospital discharge [10,11]. Social support is also highly relevant during recovery. Support from family members and loved ones may facilitate illness acceptance, reduce emotional distress, and promote adaptation to disability and engagement in rehabilitation [11]. As many stroke survivors remain dependent on others in everyday life, the consequences of stroke often extend beyond the patient and also affect caregivers [9]. In this context, caregivers play a pivotal role in supporting recovery and long-term adaptation, and a better understanding of caregiver burden and QoL may help inform tailored interventions and optimize long-term care pathways [12].

Clinical variables also appear to play an important role. Comorbid conditions may increase symptom burden, complicate rehabilitation, and worsen self-reported health status after stroke [13,14]. Among the major modifiable risk factors for stroke are hypertension, diabetes, heart disease, smoking, hyperlipidemia, alcohol consumption, substance abuse, and obesity [4,5].

Although previous studies have examined selected determinants of post-stroke QoL, most have focused on isolated clinical, functional, or psychosocial variables rather than considering functional independence, illness acceptance, perceived social support, comorbidity burden, and the timeliness of diagnosis and treatment within a single analytical framework [8,10,15,16,17,18]. Thus, the combined contribution of these factors to QoL after ischemic stroke remains insufficiently clarified. Examining these dimensions jointly may provide a more comprehensive understanding of factors associated with post-stroke QoL and may serve as a basis for future multidimensional studies in this field.

Therefore, the study aimed to analyze factors associated with the QoL in patients after ischemic stroke. Specifically, the study examined the relationships between QoL and selected sociodemographic and clinical variables, with particular emphasis on functional independence, illness acceptance, perceived social support, comorbidity status, and the timeliness of diagnosis and treatment. We hypothesized that better QoL would be associated with greater functional independence, higher illness acceptance, stronger perceived social support, fewer comorbidities, and timely diagnosis and treatment.

## 2. Materials and Methods

### 2.1. Study Design and Participants

This cross-sectional observational study was conducted among 115 patients diagnosed with ischemic stroke who were hospitalized between 1 March 2025 and 30 December 2025 in the Department of Neurology with the Stroke Unit at the S. Żeromski Specialist Hospital in Krakow, Poland. After exclusion of incomplete questionnaires, the final analysis included 100 patients. Participants were recruited consecutively from among patients admitted to the study center during the study period.

### 2.2. Eligibility Criteria

Individuals who met the inclusion criteria and provided written informed consent for participation and personal data processing were enrolled in the study.

The inclusion criteria were as follows:

(1) Confirmed diagnosis of ischemic stroke;
(2) Hospitalization in the study center during the study period;
(3) Provision of written informed consent to participate in the study;
(4) Proper completion of the study questionnaires.

The exclusion criteria were as follows:

(1) Refusal to continue participation in the study;
(2) Deterioration in health status preventing further participation;
(3) Incomplete or incorrectly completed questionnaires.

Of the 115 patients initially enrolled in the study, 15 provided incomplete questionnaires and were therefore excluded from the final analysis. In this subgroup, all patients lacked close family support and reported anxiety and concern regarding their health status after stroke. These patients were also receiving psychological support during hospitalization. Consequently, the statistical analysis was performed on data from 100 patients with complete questionnaires.

### 2.3. Ethical Approval

The study was conducted in accordance with the Declaration of Helsinki and relevant institutional and national regulations governing research involving human participants. Ethical approval was granted by the University Bioethics Committee (approval no. KBKA 34/0/2024). Written informed consent was obtained from all participants prior to inclusion in the study.

### 2.4. Data Collection

Data collection was performed at a single standardized time point during hospitalization, when the patient’s clinical condition had stabilized, and discharge home was planned. In the study group, the duration of hospitalization most commonly ranged from 7 to 10 days. The assessment was typically conducted approximately 5–7 days after stroke onset. Clinical stabilization was confirmed by neurological examination and assessment of normalized vital signs, including blood pressure, heart rate, and oxygen saturation. In addition, patients showed no aphasia or memory and concentration disturbances that could interfere with reliable questionnaire completion. All patients received rehabilitation care within the stroke unit during hospitalization. At discharge, each patient was referred for continued rehabilitation, either in an outpatient setting or in an inpatient rehabilitation center. No interventional procedures were performed, and treatment was based on pharmacological management, including intravenous thrombolysis when clinically indicated. Moreover, hemorrhagic conversion was not observed during the hospital stay. No in-hospital complications related either to stroke itself or to the treatment and hospitalization process, including aspiration pneumonia and pressure injuries, were observed in the study group.

The study was based on the diagnostic survey method and included an author-designed questionnaire together with standardized research instruments. The average time required to complete the study questionnaires was approximately 20 min. Completion of the questionnaires took place in a designated examination room within the hospital ward under conditions ensuring patient privacy. The procedure was carried out in the presence of ward nursing staff who had been previously trained in the study protocol to ensure standardized administration and reliability of the collected data. The questionnaires were completed independently by the patients. If any question was unclear or difficult to understand, the nurse provided the necessary clarification without influencing the content of the responses.

Moreover, the timeliness of diagnosis and treatment was analyzed as a dichotomous variable (Yes/No), assessed by a physician on the basis of medical documentation. Patients were classified as “Yes” if they were admitted through the Emergency Department, transferred to the Stroke Unit, and received initial treatment within 4 h of admission. All remaining patients were classified as “No”.

Additional clinical data included stroke severity assessed at admission using the National Institutes of Health Stroke Scale (NIHSS), which was routinely applied to all patients admitted to the stroke unit. Standardized research instruments were used to assess QoL, perceived social support, functional independence, and illness acceptance.

#### 2.4.1. Standardized Research Instruments

The following instruments were used in the study:

- WHOQOL-BREF (World Health Organization Quality of Life-BREF)—to assess patients’ QoL across four domains: physical, psychological, social, and environmental. Domain scores were transformed to a 0–100 scale, with higher scores indicating better QoL [6].
- Zimet’s MSPSS (Zimet’s Multidimensional Scale of Perceived Social Support)—to assess perceived social support from family, friends, and significant others [19].
- IADL (Lawton Instrumental Activities of Daily Living Scale)—to assess independence in activities of daily living [20].
- AIS (Acceptance of Illness Scale)—to assess the level of illness acceptance [21].

#### 2.4.2. Author-Designed Questionnaire

The author-designed questionnaire was used to collect selected sociodemographic and clinical data, including age, sex, place of residence, comorbidities, side of hemiplegia, self-reported health status, and the timeliness of diagnosis and treatment. In the questionnaire, age was categorized into two groups (65–75 years and over 75 years), while place of residence was classified as urban or rural. The side of stroke-related hemiplegia was classified as right- or left-sided. Comorbidity status was defined as the presence of at least one of the following conditions: hypertension, heart disease, diabetes, or obesity. Patients were classified into two groups: with comorbidities (“Yes”) and without comorbidities (“No”).

### 2.5. Analytical Framework

The analyses performed in the study included:

(1) Assessment of the normality of distribution for WHOQOL-BREF domains, IADL, AIS, and MSPSS scores;
(2) Correlation analyses between WHOQOL-BREF QoL domains and IADL, AIS, and MSPSS scores, including MSPSS subscales for family, friends, and significant others;
(3) Analysis of the relationship between IADL and AIS scores;
(4) Comparisons of WHOQOL-BREF domain scores and IADL scores according to comorbidity status;
(5) Comparisons of WHOQOL-BREF domain scores, IADL scores, and AIS scores according to the timeliness of diagnosis and treatment;
(6) Comparison of MSPSS subscale scores according to the source of perceived social support.

### 2.6. Statistical Analysis

Statistical analysis was performed using IBM SPSS Statistics software version 26 (IBM Corp., Armonk, NY, USA). Quantitative variables were presented as the mean, standard deviation (SD), and median. The normality of distribution was assessed with the Kolmogorov–Smirnov test. Because the analyzed variables deviated from normal distribution, non-parametric statistical methods were applied. Correlations between WHOQOL-BREF domains and IADL, AIS, and MSPSS scores, as well as between IADL and AIS scores, were assessed using Spearman’s rank correlation coefficient (rho). Differences between two independent groups, including comparisons according to comorbidity status and the timeliness of diagnosis and treatment, were analyzed using the Mann–Whitney U test. Differences in MSPSS subscale scores by the source of perceived social support were evaluated using the Friedman test. A *p*-value of <0.05 was considered statistically significant.

## 3. Results

### 3.1. Sociodemographic and Clinical Characteristics of the Study Group

Most participants were aged 65–75 years (68%), whereas 32% were over 75 years of age. The study group was predominantly female, with women accounting for 64% of the sample and men for 36%. More than half of the participants (59%) resided in urban areas, while 41% lived in rural areas. Comorbidities were present in 62% of the study group, whereas 38% of patients reported no comorbid conditions. Among patients with comorbidities, hypertension was the most frequent condition, accounting for 63% of cases, followed by heart disease in 20%, diabetes in 10%, and obesity in 7%. In terms of neurological deficits, right-sided hemiplegia was observed in 64% of patients, while 36% presented with left-sided hemiplegia.

According to the NIHSS, 30% of patients had mild stroke (1–4 points), 40% had moderate stroke (5–15 points), 27% had moderate-to-severe stroke (16–20 points), and 3% had severe stroke (21–42 points).

Additional timing characteristics of the study group showed that 34% of patients were admitted to the hospital within 3–4 h of stroke symptom onset, 62% within 12–24 h, and 4% between 2 and 5 days after symptom onset. Moreover, the duration of in-hospital treatment was 5–7 days in 92% of patients and 10–12 days in the remaining 8%.

### 3.2. Assessment of Distribution Normality

As shown in Table 1, all analyzed variables deviated significantly from a normal distribution. Consequently, non-parametric tests were used in further analyses.

### 3.3. Overall Characteristics of the Main Study Variables

Before subgroup comparisons and correlation analyses, the overall results indicated that QoL after ischemic stroke was closely related to functional independence, illness acceptance, perceived social support, comorbidity status, and the timeliness of diagnosis and treatment. Across the study group, better QoL was consistently associated with greater independence, higher illness acceptance, and stronger perceived social support, whereas less favorable results were observed in patients with comorbidities and in those who did not receive timely diagnosis and treatment. This overall pattern provides the context for the more detailed subgroup and correlation analyses presented below.

### 3.4. Correlations Between QoL and Functional and Psychosocial Variables

Significant positive correlations were observed between all WHOQOL-BREF domains and IADL, AIS, and MSPSS overall scores (Table 2). The strongest associations were observed between the physical and psychological domains and IADL (rho = 0.71 and rho = 0.70, respectively), whereas the weakest correlation was found between the social domain and AIS (rho = 0.50). All correlations were statistically significant (*p* < 0.001).

### 3.5. Differences in QoL According to Clinical Variables

#### 3.5.1. QoL According to Comorbidity Status

QoL scores in all WHOQOL-BREF domains differed significantly according to comorbidity status (Table 3). Patients with comorbidities (Yes) had lower scores in the physical, psychological, social, and environmental domains than patients without comorbidities (No). The most pronounced difference was observed in the physical domain (45.57 ± 18.40 vs. 82.90 ± 19.85; Z = −6.99, *p* < 0.001).

#### 3.5.2. QoL According to the Timeliness of Diagnosis and Treatment

Patients who received timely diagnosis and treatment (“Yes”) had higher WHOQOL-BREF scores across all domains than those who did not (“No”) (Table 4). The largest between-group difference was observed in the physical domain, in which the mean score was 87.75 ± 11.47 in the “Yes” group and 45.33 ± 18.95 in the “No” group (Z = −7.77, *p* < 0.001).

### 3.6. Correlation Between Functional Independence and Illness Acceptance

A statistically significant positive correlation was observed between IADL and AIS scores (rho = 0.88; *p* < 0.001; N = 100).

### 3.7. Differences in Functional Independence According to Clinical Factors

#### 3.7.1. Functional Independence According to the Presence of Comorbidities

IADL scores differed significantly according to comorbidity status (Table 5). Patients with comorbidities (“Yes”) obtained lower IADL scores than those without comorbidities (“No”). Mean values were 15.58 ± 2.41 and 24.13 ± 4.54, respectively, indicating less favorable functional independence in the comorbidity group (Z = −6.38, *p* < 0.001).

#### 3.7.2. Functional Independence According to the Timeliness of Diagnosis and Treatment

IADL scores were significantly higher in patients who received timely diagnosis and treatment (“Yes”) than in those who did not (“No”) (Table 6). The mean IADL score was 25.59 ± 2.79 in the “Yes” group and 15.35 ± 1.88 in the “No” group (Z = −7.71, *p* < 0.001).

### 3.8. Illness Acceptance According to the Timeliness of Diagnosis and Treatment

As shown in Table 7, illness acceptance was higher in patients who received timely diagnosis and treatment (“Yes”) than in those who did not (“No”). The mean AIS score was 36.53 ± 5.70 and 14.98 ± 4.12, respectively (Z = −7.60, *p* < 0.001).

### 3.9. Perceived Social Support by Source of Support

Perceived social support was comparable across the three MSPSS sources: significant others, family, and friends (Table 8). The differences between the subscale scores were not statistically significant (χ^2^ = 1.15, df = 2, *p* = 0.56, N = 100).

### 3.10. Correlations Between QoL and Sources of Perceived Social Support

All MSPSS subscales were positively correlated with all WHOQOL-BREF domains (Table 9). The strongest associations were observed for the psychological domain in relation to support from significant others (rho = 0.64), family (rho = 0.64), and friends (rho = 0.63). All correlations were statistically significant (*p* < 0.001).

## 4. Discussion

The present study provides evidence that QoL after ischemic stroke is associated with both functional and psychosocial factors. In the analyzed group, better QoL was related to greater independence, higher illness acceptance, stronger perceived social support, fewer comorbidities, and timely diagnosis and treatment. Moreover, a strong positive relationship was observed between functional independence and illness acceptance. QoL after stroke is influenced by both objective and subjective factors. Objective factors include clinical status, functional impairment, psychopathological symptoms, financial circumstances, and social contacts, whereas subjective factors relate to patients’ own perceptions of their physical, psychological, and social functioning, as well as interpersonal resources such as perceived social support [4]. Taken together, these findings indicate that post-stroke QoL is determined not only by the direct neurological consequences of stroke, but also by broader functional, psychosocial, and clinical factors.

The predominance of women in the present study group should also be interpreted in the context of age-related sex differences in stroke epidemiology. Although stroke incidence is often higher in men at younger and middle ages, women account for a substantial proportion of stroke cases at older ages because they live longer and have a higher lifetime risk of stroke [22,23,24]. Accordingly, the sex distribution observed in our sample may be related to its age structure, which included a large proportion of women aged 65 years and older, many of whom were additionally affected by hypertension.

A particularly important finding of the present study was the positive association between functional independence and QoL. Patients with higher IADL scores also obtained higher WHOQOL-BREF scores across all analyzed domains, with the strongest correlations observed for the physical and psychological domains. This result is consistent with recent evidence indicating that independence is one of the strongest determinants of post-stroke QoL. This observation is consistent with the study by Biševac et al., who showed that functional independence improved significantly during six months of postacute rehabilitation and was significantly associated with QoL [1]. Likewise, the scoping review by Gurková et al. showed that favorable functional status and greater independence in activities of daily living were among the most important factors linked to better QoL after ischemic stroke [10]. Increasing attention is being paid to patient-reported outcomes, including patients’ own perception of their health and the impact of symptoms or treatment on health-related quality of life (HRQoL). HRQoL is a multidimensional concept encompassing physical, mental, emotional, and social functioning, as well as overall well-being. Patient-reported information may be useful in comparing the effectiveness of different treatment approaches and in evaluating the effects of interventions such as occupational therapy, rehabilitation, and botulinum toxin on the QoL of stroke patients [7,25,26]. Importantly, recent evidence has also identified several predictors of HRQoL after stroke. A systematic review with meta-analysis found that fewer limitations in activities of daily living, higher functional independence, and better balance were associated with better HRQoL one year after stroke [8]. In this context, our findings reinforce the view that functional independence is not merely a rehabilitation outcome, but also a major correlate of how patients evaluate their post-stroke QoL.

The strong positive correlation between functional independence and illness acceptance is also clinically meaningful. This suggests that better functioning may facilitate psychological adaptation to the disease and to stroke-related limitations. Previous research has shown that illness acceptance in patients after stroke is associated with functional status and emotional condition during rehabilitation [15]. Lim et al. concluded that poorer function, greater disability, and depressive symptoms were associated with lower acceptance of disability after stroke, whereas better social and psychological adaptation-related factors were linked to more favorable outcomes [16]. Our results extend these observations by indicating that illness acceptance is closely linked not only to adaptation, but also to everyday independence and QoL.

Another important finding of the present study was the positive relationship between perceived social support and QoL. Positive correlations were found both for the overall MSPSS score and for each of the three sources of support analyzed separately, namely family, friends, and significant others. The strongest associations were observed for the psychological domain. Moreover, the absence of significant differences between MSPSS subscales suggests that perceived support was relatively balanced across the three main interpersonal sources. Our findings are consistent with the systematic review by Kruithof et al., which identified social support as an important determinant of HRQoL in stroke survivors [17]. Lee and Won reported that greater social support was associated with better HRQoL, with rehabilitation motivation partially mediating this relationship. These findings suggest that support from healthcare professionals and family members may contribute to improved post-stroke HRQoL by strengthening patients’ rehabilitation motivation [18]. In this context, caregivers and family members may play an important role not only in providing emotional and practical support, but also in facilitating continuity of care after hospital discharge [9]. This interpretation is also supported by observations from the excluded subgroup, in which incomplete questionnaire completion was noted among patients who lacked close family support and experienced anxiety regarding their post-stroke health status. Recovery after stroke frequently extends beyond the acute inpatient phase and may depend on access to home-based support, rehabilitation services, and structured follow-up. Current stroke guidance recommends routine follow-up of people with stroke six months after hospital discharge and annually thereafter, together with reassessment and further treatment when needed [27]. In parallel, recent evidence indicates that continuity-of-care and transitional-care interventions may improve post-stroke independence, physical functioning, self-efficacy, and QoL [28,29]. This broader post-discharge context may partly explain why the factors identified in the present study were associated with QoL after ischemic stroke.

The associations observed in the present study may also be explained by several interrelated functional, psychological, and neurobiological mechanisms. Greater functional independence may support better QoL by restoring autonomy, reducing dependence on others, and enabling broader participation in everyday and social activities. This relationship may be further shaped by emotional status, because post-stroke depression has been consistently associated with poorer outcomes across multiple functional domains, including basic and instrumental activities of daily living, physical function, and cognitive performance [30]. In addition, the effect of perceived social support on QoL may extend beyond practical assistance alone. Fong et al. showed that social support had significant positive indirect effects on physical QoL through hope and perceived stress and on psychosocial QoL through hope and depression [11]. This suggests that supportive relationships may influence QoL not only by reducing social isolation, but also by modifying emotional distress and reinforcing adaptive psychological resources. Neurobiological mechanisms may also contribute to these relationships. Recent evidence indicates that post-stroke affective disturbances are linked to a complex network of neurobiological mechanisms, including microglial activation, astrocytic responses, NF-κB-mediated inflammatory signaling, dysregulation of the hypothalamic–pituitary–adrenal axis, altered brain-derived neurotrophic factor expression, neurotransmitter imbalance, ATP-mediated signaling, and oxidative stress, all of which may adversely affect motivation, emotional regulation, and engagement in recovery [31]. Taken together, these mechanisms support the interpretation that post-stroke QoL reflects not only neurological impairment, but also the interaction between functional recovery, psychological adaptation, and social resources.

A further noteworthy finding was that patients who received timely diagnosis and treatment achieved better results in terms of QoL, functional independence, and illness acceptance. Faster time to treatment with intravenous thrombolysis has been linked to improved outcomes in acute ischemic stroke [32]. Similarly, contemporary studies of timeline-based stroke management protocols indicate that reducing treatment delays improves clinical outcomes in patients with acute ischemic stroke [33]. Although the present study did not differentiate specific acute treatment pathways in detail, this result corresponds with the broader evidence that early and effective stroke management influences long-term outcomes. Taken together, these findings support the interpretation that timely diagnosis and treatment may influence not only short-term neurological stabilization, but also later functional and patient-reported outcomes.

The present findings may also be interpreted in the context of healthcare system organization. Long waiting times for health services remain an important policy issue because they may delay the expected benefits of diagnosis and treatment and prolong patient discomfort [34]. Recent evidence indicates that access to stroke rehabilitation depends largely on availability, affordability, and awareness. Accordingly, differences between publicly and privately delivered services may influence post-stroke outcomes through their effects on access, continuity of care, and the timeliness of rehabilitation and follow-up [35]. This perspective may help explain why timely diagnosis and treatment in the present study were associated with better QoL, functional independence, and illness acceptance.

In a holistic model of care, assessment of patients’ QoL and identification of the domains most affected by stroke are important elements of both treatment and long-term care. The present study also showed that a lower burden of comorbidities, including cardiovascular disease, hypertension, diabetes, and obesity, was associated with better QoL and greater independence after ischemic stroke. This finding is clinically plausible, as the coexistence of chronic conditions may increase symptom burden, complicate rehabilitation, and reduce the patient’s ability to regain independence. In the subgroup of patients with comorbidities, hypertension was the most frequent condition and was present in 63% of cases. Similar findings were reported by Trojanowska et al., who observed hypertension in 92% of patients [36], and by Jaracz and Kozubski, who found that it affected more than half of their study population [37,38]. Heinze et al. demonstrated that a higher burden of comorbid conditions at admission was independently associated with worse self-reported physical and mental health three months after acute ischemic stroke [13]. Likewise, multimorbidity is common among stroke survivors and has been associated with less favorable medium- and long-term outcomes [14].

From a clinical perspective, the present findings support a comprehensive approach to post-stroke care. QoL appears to depend not only on neurological impairment itself, but also on the patient’s ability to regain independence, adapt psychologically to illness, and rely on stable social support. Therefore, rehabilitation programs should not focus exclusively on physical recovery, but should also include interventions that strengthen patient motivation, social support, and coping with long-term disability. Such an approach may be especially important in older stroke survivors, in whom functional limitations and dependence on others frequently persist beyond the acute phase.

The results of the present study should be interpreted in light of several methodological limitations. First, due to the cross-sectional design, the observed relationships should be interpreted as associations rather than causal relationships. Although better QoL was associated with greater functional independence, higher illness acceptance, stronger perceived social support, fewer comorbidities, and timely diagnosis and treatment, it cannot be determined whether these factors influenced QoL, whether better QoL contributed to more favorable functional and psychosocial outcomes, or whether these relationships were bidirectional. Second, the study was conducted in a single center and included a relatively small sample, which limits the representativeness of the study population and reduces the generalizability of the findings to broader populations of patients after ischemic stroke. In addition, the results may have been influenced by the organization of care at the study center. Third, the analysis was based largely on self-reported measures of QoL, social support, and illness acceptance, which may be influenced by temporary emotional state or response style. Future multicenter prospective studies are needed to confirm these relationships and to further examine their clinical significance. Nevertheless, the study provides clinically relevant evidence on the functional, psychosocial, and clinical correlates of QoL after ischemic stroke.

Overall, the present results suggest that better post-stroke QoL is associated with greater functional independence, stronger social support, higher illness acceptance, and timely diagnosis and treatment. These findings underline the need for multidimensional post-stroke care models that integrate acute treatment, rehabilitation, and psychosocial support in order to improve long-term patient outcomes.

## 5. Conclusions

QoL after ischemic stroke was associated with functional independence, illness acceptance, perceived social support, comorbidity status, and the timeliness of diagnosis and treatment. Higher QoL scores were observed in patients with greater independence, higher illness acceptance, stronger perceived social support, fewer comorbidities, and timely diagnosis and treatment. Functional independence was also strongly positively associated with illness acceptance. These findings suggest that QoL after ischemic stroke is related not only to clinical status, but also to functional and psychosocial factors. The present findings also indicate that the assessment of QoL, independence, social support, and illness acceptance may be useful in the comprehensive evaluation of patients after ischemic stroke. However, given the single-center cross-sectional design and relatively small sample, the results should be interpreted with caution and require confirmation in larger multicenter studies.

## Figures and Tables

**Table 1:** Distribution normality of the analyzed variables.

Variable	N	*p*
WHOQOL-BREF domain 1 (physical)	100	0.02
WHOQOL-BREF domain 2 (psychological)	100	<0.001
WHOQOL-BREF domain 3 (social)	100	<0.001
WHOQOL-BREF domain 4 (environmental)	100	<0.001
IADL	100	<0.001
AIS	100	<0.001
MSPSS friends	100	<0.001
MSPSS family	100	<0.001
MSPSS significant others	100	<0.001

Abbreviations: N—number of participants; *p*—*p*-values.

**Table 2:** Correlations between WHOQOL-BREF domains and IADL, AIS, and MSPSS overall scores.

WHOQOL-BREF Domain	Statistic	IADL	AIS	MSPSS Overall
Physical	rho	0.71	0.61	0.58
	*p*	<0.001	<0.001	<0.001
	N	100	100	100
Psychological	rho	0.70	0.67	0.63
	*p*	<0.001	<0.001	<0.001
	N	100	100	100
Social	rho	0.55	0.50	0.57
	*p*	<0.001	<0.001	<0.001
	N	100	100	100
Environmental	rho	0.59	0.57	0.59
	*p*	<0.001	<0.001	<0.001
	N	100	100	100

Abbreviations: rho—Spearman’s rank correlation coefficient; *p*—*p*-values; N—number of participants.

**Table 3:** WHOQOL-BREF domain scores according to comorbidity status.

WHOQOL-BREF Domain	Comorbidities	Mean ± SD	Median	N	Z	*p*
Physical	Yes	45.57 ± 18.40	50.00	62	−6.99	<0.001
	No	82.90 ± 19.85	83.33	38		
Psychological	Yes	19.73 ± 9.59	19.23	62	−6.55	<0.001
	No	44.43 ± 14.69	50.00	38		
Social	Yes	50.27 ± 11.09	50.00	62	−6.53	<0.001
	No	76.32 ± 16.61	83.33	38		
Environmental	Yes	56.70 ± 18.28	53.13	62	−5.70	<0.001
	No	82.65 ± 20.73	90.63	38		

Abbreviations: SD—standard deviation; N—number of participants; Z—standardized test statistic for the Mann–Whitney U test; *p*—*p*-values.

**Table 4:** WHOQOL-BREF domain scores according to the timeliness of diagnosis and treatment.

WHOQOL-BREF Domain	Timeliness of Diagnosis and Treatment	Mean ± SD	Median	N	Z	*p*
Physical	Yes	87.75 ± 11.47	87.50	34	−7.77	<0.001
	No	45.33 ± 18.95	50.00	66		
Psychological	Yes	47.85 ± 11.64	53.85	34	−7.30	<0.001
	No	19.46 ± 9.12	19.23	66		
Social	Yes	79.41 ± 14.83	83.33	34	−6.96	<0.001
	No	50.25 ± 10.64	50.00	66		
Environmental	Yes	88.24 ± 14.72	90.63	34	−6.94	<0.001
	No	55.40 ± 17.89	50.00	66		

Abbreviations: SD—standard deviation; N—number of participants; Z—standardized test statistic for the Mann–Whitney U test; *p*—*p*-values.

**Table 5:** IADL scores according to comorbidity status.

Comorbidities	Mean ± SD	Median	N	Z	*p*
Yes	15.58 ± 2.41	15.00	62	−6.38	<0.001
No	24.13 ± 4.54	26.00	38		
Total	18.83 ± 5.35	16.00	100		

Abbreviations: SD—standard deviation; N—number of participants; Z—standardized test statistic for the Mann–Whitney U test; *p*—*p*-values.

**Table 6:** IADL scores according to the timeliness of diagnosis and treatment.

Timeliness of Diagnosis and Treatment	Mean ± SD	Median	N	Z	*p*
Yes	25.59 ± 2.79	27.00	34	−7.71	<0.001
No	15.35 ± 1.88	15.00	66		
Total	18.83 ± 5.35	16.00	100		

Abbreviations: SD—standard deviation; N—number of participants; Z—standardized test statistic for the Mann–Whitney U test; *p*—*p*-values.

**Table 7:** AIS scores according to the timeliness of diagnosis and treatment.

Timeliness of Diagnosis and Treatment	Mean ± SD	Median	N	Z	*p*
Yes	36.53 ± 5.70	38.00	34	−7.60	<0.001
No	14.98 ± 4.12	14.00	66		
Total	22.31 ± 11.28	16.00	100		

Abbreviations: SD—standard deviation; N—number of participants; Z—standardized test statistic for the Mann–Whitney U test; *p*—*p*-values.

**Table 8:** MSPSS subscale scores according to the source of perceived social support.

		MSPSS Significant Others	MSPSS Family	MSPSS Friends
**Statistics**	Mean ± SD	15.88 ± 8.22	16.00 ± 7.99	15.86 ± 7.79
	Median	15.00	13.50	12.00
	Minimum	5	5	5
	Maximum	28	28	28

Abbreviations: SD—standard deviation.

**Table 9:** Correlations between WHOQOL-BREF domains and MSPSS subscale scores.

WHOQOL-BREF Domain	Statistic	MSPSS Friends	MSPSS Family	MSPSS Significant Others
Physical	rho	0.61	0.58	0.61
	*p*	<0.001	<0.001	<0.001
	N	100	100	100
Psychological	rho	0.63	0.64	0.64
	*p*	<0.001	<0.001	<0.001
	N	100	100	100
Social	rho	0.59	0.58	0.55
	*p*	<0.001	<0.001	<0.001
	N	100	100	100
Environmental	rho	0.61	0.59	0.58
	*p*	<0.001	<0.001	<0.001
	N	100	100	100

Abbreviations: rho—Spearman’s rank correlation coefficient; *p*—*p*-values; N—number of participants.

## Data Availability

The original contributions presented in this study are included in the article. Further inquiries can be directed to the corresponding author.

## Abbreviations

The following abbreviations are used in this manuscript:

AIS	Acceptance of Illness Scale
CNS	Central Nervous System
CVD	Cardiovascular Disease
GBD	Global Burden of Disease
HRQoL	Health-Related Quality of Life
IADL	Instrumental Activities of Daily Living
NIHSS	National Institutes of Health Stroke Scale
MSPSS	Multidimensional Scale of Perceived Social Support
QoL	Quality of Life
WHO	World Health Organization
WHOQOL-BREF	World Health Organization Quality of Life-BREF

## References

[1] Bisevac E., Safiye T., Mahmutovic E. (2025). Cognitive Status and Quality of Life of Post-Stroke Patients: Evidence from Serbia. VSP.

[2] Feigin V.L., Brainin M., Norrving B., Martins S.O., Pandian J., Lindsay P., Grupper M., Rautalin I. (2025). World Stroke Organization: Global Stroke Fact Sheet 2025. Int. J. Stroke.

[3] Sacco R.L., Kasner S.E., Broderick J.P., Caplan L.R., Connors J.J., Culebras A., Elkind M.S.V., George M.G., Hamdan A.D., Higashida R.T. (2013). An Updated Definition of Stroke for the 21st Century: A Statement for Healthcare Professionals From the American Heart Association/American Stroke Association. Stroke.

[4] Divyanshi, Mahto P., Sharma C.K., Gupta S., Zafar S., Agarwal S. (2025). Effect of Pelvic Proprioceptive Neuromuscular Facilitation on Balance, Postural Sway and Quality of Life in Chronic Stroke Patients: A Case Series. J. Clin. Diagn. Res..

[5] Murphy S.J.X., Werring D.J. (2020). Stroke: Causes and Clinical Features. Medicine.

[6] World Health Organization, Division of Mental Health and Prevention of Substance Abuse (1998). WHOQOL User Manual.

[7] Butsing N., Voss J.G., Keandoungchun J., Thongniran N., Griffin M.T.Q. (2025). Changes of Health-Related Quality of Life within 6 Months after Stroke by Clinical and Sociodemographic Factors. Sci. Rep..

[8] Silva A.C., Menezes K.K.P., Scianni A.A., Avelino P.R., Faria C.D.C.d.M. (2024). Predictors of Health-Related Quality of Life One Year after Stroke: A Systematic Review with Meta-Analysis. Int. J. Rehabil. Res..

[9] Chen H., Makhdzir N., Loh W.C., Wang L. (2025). Quality of Life of Family Caregivers of Stroke Patients: A Systematic Review of Qualitative Research. Pak. J. Med. Sci..

[10] Gurková E., Štureková L., Mandysová P., Šaňák D. (2023). Factors Affecting the Quality of Life after Ischemic Stroke in Young Adults: A Scoping Review. Health Qual. Life Outcomes.

[11] Fong T.C.T., Lo T.L.T., Ho R.T.H. (2022). Indirect Effects of Social Support and Hope on Quality of Life via Emotional Distress among Stroke Survivors: A Three-Wave Structural Equation Model. Front. Psychiatry.

[12] Pucciarelli G., Ausili D., Galbussera A.A., Rebora P., Savini S., Simeone S., Alvaro R., Vellone E. (2018). Quality of Life, Anxiety, Depression and Burden among Stroke Caregivers: A Longitudinal, Observational Multicentre Study. J. Adv. Nurs..

[13] Heinze M., Lebherz L., Rimmele D.L., Frese M., Jensen M., Barow E., Lettow I., Kriston L., Gerloff C., Härter M. (2022). Higher Comorbidity Burden Is Associated with Lower Self-Reported Quality of Life after Stroke. Front. Neurol..

[14] Downer M.B., Luengo-Fernandez R., Binney L.E., Gutnikov S., Silver L.E., McColl A., Rothwell P.M. (2024). Association of Multimorbidity with Mortality after Stroke Stratified by Age, Severity, Etiology, and Prior Disability. Int. J. Stroke.

[15] Guzek Z., Kowalska J. (2020). Analysis of the Degree of Acceptance of Illness Among Patients After a Stroke: An Observational Study. Clin. Interv. Aging.

[16] Lim M.J.R., Tan J., Neo A.Y.Y., Ng B.C.J., Asano M. (2024). Acceptance of Disability in Stroke: A Systematic Review. Ann. Phys. Rehabil. Med..

[17] Kruithof W.J., van Mierlo M.L., Visser-Meily J.M.A., van Heugten C.M., Post M.W.M. (2013). Associations between Social Support and Stroke Survivors’ Health-Related Quality of Life—A Systematic Review. Patient Educ. Couns..

[18] Lee Y., Won M. (2022). Mediating Effects of Rehabilitation Motivation between Social Support and Health-Related Quality of Life among Patients with Stroke. Int. J. Environ. Res. Public Health.

[19] Zimet G.D., Dahlem N.W., Zimet S.G., Farley G.K. (1988). The Multidimensional Scale of Perceived Social Support. J. Personal. Assess..

[20] Lawton M.P., Brody E.M. (1969). Assessment of Older People: Self-Maintaining and Instrumental Activities of Daily Living. Gerontologist.

[21] Czerw A., Religioni U., Szumilas P., Sygit K., Partyka O., Mękal D., Jopek S., Mikos M., Strzępek Ł. (2022). Normalization of the AIS (Acceptance of Illness Scale) Questionnaire and the Possibility of Its Use among Cancer Patients. Ann. Agric. Environ. Med..

[22] Vyas M.V., Silver F.L., Austin P.C., Yu A.Y.X., Pequeno P., Fang J., Laupacis A., Kapral M.K. (2021). Stroke Incidence by Sex Across the Lifespan. Stroke.

[23] Dawson J., MacDonald A. (2023). Sex and Hypertensive Organ Damage: Stroke. J. Hum. Hypertens..

[24] Hanna M., Wabnitz A., Grewal P. (2024). Sex and Stroke Risk Factors: A Review of Differences and Impact. J. Stroke Cerebrovasc. Dis..

[25] Caty G.D., Detrembleur C., Bleyenheuft C., Deltombe T., Lejeune T.M. (2008). Effect of Simultaneous Botulinum Toxin Injections Into Several Muscles on Impairment, Activity, Participation, and Quality of Life Among Stroke Patients Presenting with a Stiff Knee Gait. Stroke.

[26] Caty G.D., Detrembleur C., Bleyenheuft C., Deltombe T., Lejeune T.M. (2009). Effect of Upper Limb Botulinum Toxin Injections on Impairment, Activity, Participation, and Quality of Life Among Stroke Patients. Stroke.

[27] Intercollegiate Stroke Working Party (2023). National Clinical Guideline for Stroke, 2023.

[28] Premnazeer M., Munce S.E.P., Bayley M.T., Kokorelias K.M., Gill M., Scali O.L., Cameron J.I. (2025). Continuity of Care Interventions for People with Stroke and Their Caregivers: A Scoping Review Protocol. PLoS ONE.

[29] Veronese M., Vellone E., Alvaro R., Pucciarelli G. (2025). The Transitional Care from Hospital to Home for Stroke Survivors and Their Caregivers: A Systematic Review. J. Vasc. Nurs..

[30] Butsing N., Zauszniewski J.A., Ruksakulpiwat S., Griffin M.T.Q., Niyomyart A. (2024). Association between Post-Stroke Depression and Functional Outcomes: A Systematic Review. PLoS ONE.

[31] Feng X., Ma X., Li J., Zhou Q., Liu Y., Song J., Liu J., Situ Q., Wang L., Zhang J. (2024). Inflammatory Pathogenesis of Post-Stroke Depression. Aging Dis..

[32] Man S., Solomon N., Mac Grory B., Alhanti B., Uchino K., Saver J.L., Smith E.E., Xian Y., Bhatt D.L., Schwamm L.H. (2023). Shorter Door-to-Needle Times Are Associated With Better Outcomes After Intravenous Thrombolytic Therapy and Endovascular Thrombectomy for Acute Ischemic Stroke. Circulation.

[33] Li Y.-F., Feng G.-Q., Ao Y.-P., Gu B., Wang J., Xiao A.-F., You J.-Q. (2025). Impact of Timeline-Based Management on Treatment Efficiency and Outcomes in Acute Ischemic Stroke: A Quasi-Experimental Study. Ann. Med..

[34] OECD (2020). Waiting Times for Health Services: Next in Line.

[35] Gandhi D.B.C., Baggio J.A.O., D’Souza J.V., Urimubenshi G., Vijayanand P.J. (2024). Global Access to Stroke Rehabilitation: A Narrative Synthesis of Comparative Highlights. Stroke.

[36] Trojanowska A. (2011). Znaczenie Badań Nad Jakością Życia w Medycynie. Pol. J. Public Health.

[37] Jaracz K., Kozubski W. (2001). Jakość Życia Po Udarze Mózgu. Część I–Badanie Prospektywne. Udar Mózgu.

[38] Jaracz K., Kozubski W. (2002). Jakość Życia Chorych Po Udarze Mózgu w Świetle Badań Empirycznych. Aktual. Neurol..

